# Effective altruism, technoscience and the making of philanthropic value

**DOI:** 10.1080/03085147.2024.2439715

**Published:** 2025-02-07

**Authors:** Apolline Taillandier, Neil Stephens, Samantha Vanderslott

**Affiliations:** a Apolline Taillandier (corresponding author), Leverhulme Centre for the Future of Intelligence, Department of Politics and International Studies, Newnham College, University of Cambridge, Cambridge, United Kingdom. E-mail: amit3@cam.ac.uk; b Neil Stephens, School of Social Policy and Society, University of Birmingham, Birmingham, United Kingdom. E-mail: n.stephens@bham.ac.uk; c Samantha Vanderslott, Oxford Vaccine Group, Department of Paediatrics, University of Oxford, Oxford, United Kingdom and NIHR Oxford Biomedical Research Centre, Oxford, United Kingdom. E-mail: samantha.vanderslott@paediatrics.ox.ac.uk

**Keywords:** effective altruism, philanthropy, sociotechnical imaginaries, neglected tropical diseases, cultured meat, AI safety

## Abstract

A recent philanthropic movement with advocates among high-profile tech entrepreneurs and philosophers, effective altruism (EA) has been widely disparaged for its flawed moral philosophy and conservative political implications. As philanthropic practice, however, it has been seldom studied. In this paper, we argue that claims to technoscientific expertise are central to how EA actors understand, legitimize, and take part in the production of philanthropic value. We analyze their practices of categorization, ranking and measurement as well as underlying technoscientific imaginaries and moral views through comparing three areas of EA intervention: neglected tropical diseases, cultured meat, and AI safety. We show how EA involves various and contested ambitions to direct knowledge production and redraw the boundaries of expert communities, shedding light on the centrality of technoscience in philanthropists’ worldmaking ambitions.

## Introduction

Little over 15 years old, effective altruism (EA) aims to maximize ‘the most good’ (MacAskill, 2015) one can do through philanthropy. Its main principle, developed in popular books such as *The most good you can do* (Singer, 2015), *The precipice* (Ord, 2020), and *What we owe the future* (MacAskill, 2022), is that individuals and non-profits should decide where and how to donate or volunteer based on scientific evidence, to ensure that their money or time contributes to maximizing global welfare. In the words of philosopher and advocate Toby Ord, EA is ‘to the pursuit of the good [what] the scientific revolution was to the pursuit of truth’ (Future of Life Institute, 2016). This ambition is not new. Claims about the morality of capitalism have a long history, and the search for ‘efficiency’ in doing good can be seen as a key tenet of modern philanthropy (Boston Review Forum, 2015; Brest, 2020). Philanthropists have long sought to impose their conception of the good by training political and economic elites (Guilhot, 2007) or actively shaping new fields of expertise (Bartley, 2007). EA organizations in part further this tradition by funding research related to the philanthropic causes they consider to be the most cost-efficient. With high-profile tech entrepreneurs advocating ‘earning to give’ or ‘longtermism’ (Bajekal, 2022; MacAskill, 2022), EA recently received sustained public attention. It has been deemed the new ‘secular religion’ of Silicon Valley elites (Wenar, 2024; see also Gebru & Torres, 2024).

EA, not unlike the Silicon Valley social milieu (Alexandre, 2022; Turner, 2006), is characterized by the coexistence of strong symbolic and material hierarchies with a progressive and egalitarian ethos. But rather than a Californian phenomenon, it is a transnational complex with advocates spanning the United States and Europe. Known as ‘EAs’, these include wealthy entrepreneurs such as the recently disgraced Sam Bankman-Fried, high-profile philosophers based at elite universities in the United States, the United Kingdom and Australia, and advocates among graduate students and early career researchers (Hyatt, 2022; Rubenstein, 2016). Typically young, well-educated and trained in the West, EAs are predominantly aged between 25 and 34, male, white, non-religious, politically on the left or centre left, living in the United States or Britain; 46 per cent are vegan, more than 90 per cent have a higher-education degree, and 20 per cent attend one of the top 20 universities in the world (Dullaghan, 2019; see also Moss, 2020). Organizations committed to EA principles include private foundations – such as San Francisco-based Open Philanthropy and Good Ventures, both set up by Facebook co-founder and billionaire Dustin Moskovitz and former journalist Cari Tuna – and non-profits – such as the charity evaluator GiveWell created by former hedge-fund managers Elie Hassenfeld and Holden Karnofsky, and the Oxford-based Centre for Effective Altruism led by philosophers Toby Ord and William MacAskill. These organizations evaluate, recommend and fund charities on the basis of the number of lives improved per dollar donated: during 2018, GiveWell claimed to have directed more than $140 million to ‘highly effective charities’ through grants and by influencing individual donations (GiveWell, 2018). EA’s public facing initiatives include the website ‘80,000 Hours’ advising on ‘high-impact careers’ and the ‘Giving What We Can Pledge’ campaign to encourage ‘the richest 2 per cent globally’ to donate 10 per cent of their income to effective non-profits (Giving What We Can, 2024). EAs also meet at annual conferences, share houses, organize informal meetups and interact through online forums and blogs.

A growing scholarship explores EA’s ideological and narrative dimensions. Philosophers and political theorists have discussed at length how its ambition to increase global welfare recasts long-standing arguments about the source, nature and scope of moral obligations (Adams *et al*., 2023; McMahan, 2016; Parfit, 2015). They usually trace its broad inspiration in utilitarianism, a strand of moral philosophy concerned with maximizing individual or collective utility or welfare: as political theorist Jennifer Rubenstein (2016) put it, ‘the shadow of utilitarianism looms large’ over EA (p. 513). Associated with analytic philosophers such as Peter Singer, William MacAskill, Toby Ord and Hilary Greaves, it recently became an object of philosophical inquiry (Greaves & Pummer, 2019; Kumar, 2020). Critics have found EA philanthropy to be mostly conservative: despite its ‘willingness to challenge the status quo’ (Future of Life Institute, 2016), it dilutes philosophical ideas into ‘an empowering investment opportunity’ (Srinivasan, 2015), focuses on individual donations rather than political advocacy and institutional action aiming at structural change (Blunt 2022; Herzog, 2016; Lechterman, 2022; Saunders-Hastings, 2019), and assumes a ‘rescue’ idea of philanthropy with deep depoliticizing and paternalist effects (Deveaux, 2021, pp. 48–79). Sociologists have described EA as the latest iteration of ‘philanthrocapitalism’ – a mode of philanthropic action privileging donations to for-profits and interventions into domains with high expected returns, manifesting the extension of market-based methods and private funding priorities to traditional spheres of state intervention, such as health and development (Eikenberry & Mirabella, 2017; McGoey & Thiel, 2018). While evidence-based philanthropy is not a new phenomenon, philanthrocapitalism also manifests the incursion of ‘high-net-worth individuals’ from the technology sector in a space traditionally dominated by large foundations (Depecker *et al*., 2018; Haydon *et al*., 2021; McGoey 2012a, 2015). EA actors themselves, calling for alliances with other actors in the charity sector, have noted the tensions arising from such reconfigurations of the philanthropic field (Gabriel 2017). Our study contributes to clarifying how structural transformations in philanthropy affect giving practices, institutions and rhetoric.

In this paper, we argue that EA is best understood as a form of technoscientific worldmaking. EAs fund technological projects with high expected moral value, contest the legitimacy of established knowledge production, and aim to reshape whole fields of expertise. In doing so, they both define global subjects – the global poor, sentient beings or future people – and what counts as global problems. This dimension of EA philanthropy has been little analysed, even while EAs have been adamant to support and orient academic research to meet their goals. During 2018, Open Philanthropy recommended over $170 million donations in grants to organizations researching ‘potential risks of advanced AI’, ‘biosecurity and pandemic preparedness’, criminal justice reform, and animal welfare (Open Philanthropy, 2022 [2018]), and in 2024 it partnered with the UK Department for Science, Innovation and Technology on a £5 million grants programme to support projects on ‘more effective ways of conducting and supporting research and development’ (UK Research and Innovation, 2024). Our analysis concentrates on three areas of EA intervention: (1) the fight against neglected tropical diseases (NTDs) to reduce mortality in the Global South, (2) the development of cultured meat to reduce animal suffering, and (3) the advancement of artificial intelligence (AI) safety to reduce risks of extinction to future humanity. In each case, we show how EAs translate philosophical views into actionable tools including rankings, forecasts and assessments of efficiency, and how they seek to maximize moral and economic value through technoscientific innovation. By studying EA in the making, we offer an empirical complement to the ideology critique of philanthrocapitalist logics.

This analysis brings together three strands of scholarship. We draw, firstly, on the study of the ‘politics of measurement’ (Lupton, 2013). As sociologists of quantification have extensively argued, both quantitative and systematic qualitative data collection aim to stabilize facts (i.e. some objective account of reality), and they are shaped by and contribute to materializing values and hierarchies (Best, 2012; Bowker & Star, 1999; Desrosières, 2002; Hacking, 1991; Porter, 1997). Rankings, for instance, enable action under uncertainty (Esposito & Stark, 2019). EA philanthropy materializes through what we call *philanthropic metric work* (on metric work, see Adams, 2016), a set of practices involving the development, codification and stabilization of concrete instruments of commensuration, classification and ranking to assess the effectiveness of charities and donations. Secondly, we expand on the analysis of ‘sociotechnical imaginaries’, the ‘collectively held and performed’ interplay between visions of technoscientific futures and visions of desirable social and moral orders (Jasanoff & Kim, 2015, p. 15). Philanthropists, like other economic actors, produce both an idea of the social and the tools to intervene into it. Our study shows how EAs in different settings perform distinct visions of desirable technology and society. Thirdly, we draw on the economic sociology literatures evidencing the role of technoscientific futures in driving innovation (Borup *et al*., 2006; Joly, 2010) and securing financial value (Beckert & Bronk, 2018; Muniesa, 2017). As we show across our cases, technoscientific promises, expectations of future technological capability, and claims to expertise play a central role in how EAs define and justify philanthropic investment.

Philanthropic value creation, we argue further, is a contested process. A key EA problem has been to assess not only the effectiveness of individual donations, but also which ‘causes’ are the most important, tractable and neglected, and should therefore be prioritized. Typically referred to as ‘cause prioritization’, this debate has aimed to delimit and rank areas of intervention and opportunities for investment – notably comparing between extreme poverty alleviation, reduction of animal suffering, mitigation of extinction risks related to future technology, and support to EA recruitment and organizing (e.g. Muehlhauser, 2015a). Many EA organizations shifted their focus from global poverty to AI risk in recent years, a turn that critics have analyzed as a departure from the movement’s original commitment to rationality and a sign of unwarranted futuristic speculation (Schuster & Woods, 2021, pp. 45–78; Srinivasan, 2015). In our analysis, these debates evidence the contested character of EA practice rather than its fundamental epistemological flaw or inconsistency. EA interventions are shaped by two opposing views of philanthropy: an ideal of decentralized philanthropy premised upon visions of disruptive technological breakthroughs and radical social change, and an ideal of technocratic philanthropy assuming a linear vision of technology-enabled moral progress. Justifying different and partly incompatible investment strategies, expert claims and metric work perform rather than neutralize this tension. The study of EA brings to light the conflicted politics of philanthrocapitalism.^1^

## Methods

This paper is the result of a four-year collaboration between the three authors. It brings together three qualitative studies originally conducted independently. The first study examined the policy history of NTDs, and the advocacy campaign to raise their profile, through documentary analysis of journal articles, books, media and policy documents, and 55 interviews with scientists, policy officials and NGO workers, from an initial purposeful sample expanded using snowballing technique (2013–2017) (Vanderslott, 2021). The second study focused on cultured meat communities and involved documentary analysis of texts produced by media actors and both cultured meat supporters and detractors, 76 interviews with scientists, funders and proponents of cultured meat, and ethnographic observations (2010–2023) (Stephens 2013, Stephens *et al*., 2019). The third study traced the political history of transhumanism from the 1970s onwards through published and archival materials and 46 interviews with actors in EA, existential risk studies and transhumanism in the San Francisco Bay Area and in Oxford, Cambridge and London (2016–2021) (Taillandier, 2021a). The analysis in this paper was conducted through a workshop series and recurrent exchanges between the three authors, all of whom conducted additional text analysis and interviews for this paper, respectively: five additional interviews in NTDs, 20 in cultured meat and 15 in AI safety. Analytical themes were generated and interrogated collectively across this body of work and are reported below.

## Case-study 1: NTDs and the remaking of neglect

NTDs have been a sustained topic of EA interest. Currently, the World Health Organization lists 20 NTDs, including dengue fever, leprosy and schistosomiasis. The label ‘neglected’ was applied to this disease group in the early 2000s in contrast with the ‘big three’ diseases of HIV/AIDS, tuberculosis and malaria in order to attract funding from international donors, and it has subsequently received growing policy attention (Vanderslott, 2019). NTDs are clustered together under one title due to their shared status in global health politics rather than a shared biological mechanism. They are diseases of poverty, collectively affecting over one in four people in the world. EAs commonly describe NTDs as a low-hanging fruit in global health, an area they could meaningfully impact through supporting the deployment of existing solutions. As one EA said, ‘I think that, broadly speaking, when EA started, [it] was to a large extent about scaling up existing global poverty interventions, which it was widely believed that we had strong evidence for’. EA interventions in global health often entail scaling-up low-technology and tried-and-tested interventions, such as bed-nets and deworming pills.

EA organizations typically rank causes based on criteria of ‘Importance, Tractability and Neglectedness’ (ITN). This ITN framework aims to determine the scale of a problem, the possibility of meaningfully addressing it, and the significance of existing philanthropic efforts. This typically entails evaluating the cost-effectiveness of research into NTDs. Max Dalton, director of the Centre for Effective Altruism until 2023, conducted quantitative research to ‘assess the impact of an additional dollar given to a research programme, rather than the average impact of all of the dollars that fund a research programme’ (Dalton, 2015, p. 4). Dalton’s work, which emphasized the incremental contribution of the ‘next’ donation and the value of the ITN framework to assess it and was used by Giving What We Can to justify non-profit ranking, is an example of what we call philanthropic metric work. It aimed to identify how to maximize the impact of each individual donation, and assumed that well-informed, strategic donations could make a disproportionately large contribution to global welfare and poverty reduction.

Dalton’s assessment illustrates how EAs redefine ‘neglect’ in the context of tropical diseases. While NGOs have defined NTDs in contrast with HIV/AIDS, tuberculosis and malaria, Dalton collated both categories in his calculations: as he explained, although the ‘big three’ are less underfunded than NTDs, they ‘fulfill all of the criteria for NTDs’ (Dalton, 2015, p. 5), especially as they remain underfunded compared to other diseases. Dalton mobilized an alternative definition of ‘neglect’ to that commonly used by NTD scientists, activists and policymakers: in opposition to the NTD community that sought to assert difference based upon profile, his definition bound together a broader group of tropical diseases. The same is evident at GiveWell. In 2017, the organization listed both the Malaria Consortium and the Against Malaria Foundation as key charities; the rationale for including malaria being its characterization as a high-mortality disease, the mortality of which can be lessened by low-cost interventions such as anti-malaria nets (GiveWell, 2017). This reinforces the view that ‘neglect’ should be balanced with cost-effectiveness calculations and contributes to making poverty-related diseases commensurable in a way that challenges dominant policy categorizations. By developing their own metrics, EAs contest the monopoly of NTD experts in defining what constitutes neglect. In doing so, they assert their own way of establishing global health priorities.

Philanthropic metric work, though, can prove difficult. EAs, while typically prioritizing their own rankings above those commonly in use within the expert communities they interact with, also recognize that their own expertise lacks established credentials in medicine or policymaking. Dalton, for example, studied philosophy, politics and economics before conducting his study of tropical diseases, and had no prior experience of working on NTDs. Some interviewees questioned the epistemic value of such outsider’s perspective. As one asked, ‘how much can a smart but non-expert person read stuff and come to valid conclusions, and how much do you need actual expertise?’. Another interviewee provided further context: ‘the main advocates for cause areas, none of them have had an academic or professional background in these areas. GiveWell was started by hedge fund people. Giving What We Can was started by philosophers. [AI safety researcher] Eliezer Yudkowsky dropped out of high school. So, *everybody is self-taught*. And I think people have rightly criticized us for not having a professional background. I think people [in EA] have made up for that to a large degree’ (emphasis added). EA organizations also commonly point out the limits of their own efforts in assessing efficiency. In 2012, Giving What We Can revised its original 2009 estimate of the cost-effectiveness of deworming treatments, one of the leading interventions used by the major NTD charities (Cotton-Barratt, 2012). The revision came after significant errors in the estimates of the Disease Control Priorities Project that informed the original figure were identified by the British charity Cochrane Review, indicating that deworming treatments were less effective than first thought (Cotton-Barratt, 2012). For a period, one of the key charities – Schistosomiasis Control Initiative – was somewhat ambiguously labelled ‘Top Charities without capacity to use new donations effectively at this time’ (GiveWell, 2019). Following this recategorization, the organization stopped being one of GiveWell’s top-rated charities in 2022 (GiveWell, 2022).

Interviewees typically questioned the precision and reliability of their own metrics. One commented on the shifting status of the Schistosomiasis Control Unit and malaria charities: ‘I give most of my donations to Against Malaria, which is still considered *the* EA charity. And Schistosomiasis Control Initiative. It’s considered, now, a safe bet … ’. Discussing ‘longtermism’ – the view that long-term future welfare should weigh significantly more in resource allocation than it currently does – the interviewee explained that donating to charities fighting malaria ‘[is] not the intervention with the highest expected value, but, honestly, with a longtermist view, I don’t know if big, expected value really works, *because they are kind of like magic numbers’* [emphasis added]. Another noted the difficulty of grounding expected utility measures: ‘I think, basically over time, EAs have started to look at things with more error bars around them and expect that the numbers are less firm than they initially seemed’. Commenting further on the ‘burden’ of measurement and concerned that expectations of conclusive results are not always met, one interviewee told us: ‘you get these RCTs [randomized controlled trials] done, it costs you so much money and effort. And then it’s like there’s barely any difference with your intervention. You were like, based on other evidence, do you really think it works? And it’s just maddening. And EA is committed to being about evidence. And I just think people have gotten tired of that level of rigour, frankly’. Acknowledging the limits of metrics is part and parcel of EA’s metric work.

In comparison to cultured meat and AI safety, interactions between EAs supporting intervention into NTDs and the scientists conducting research on tropical disease are limited. Alan Fenwick, a professor of tropical parasitology at Imperial College London who founded the Schistosomiasis Control Initiative in 2002, has pointed out the unexpected income stream for donations from the public and high-net-worth individuals since Giving What We Can and GiveWell both listed the Schistosomiasis Control Initiative as a top charity in 2011 (Fenwick, 2017; GiveWell, 2011). In the following years, while he attended EA events to further promote his work, EAs rarely engaged in developing innovations with him or other scientific researchers. Instead, they sought to funnel support to established but underfunded interventions. As one explained, ‘The EA approach isn’t really about development or new treatments or anything. So, it doesn’t really overlap with what most medical or academic people involved with those NTDs would be doing. It’s more like a supply line thing … just picking something where there’s low-hanging fruit. There’s not really a question, how do you treat this? It’s more like, how do you coordinate? … Definitely, EA is more involved with public health people, than in medicine’. This suggests a logistically driven vision of public health that requires distributing known treatments more effectively or fairly, rather than innovating to produce new ones.

In the case of NTDs, EAs identify problems and priorities differently both to the aid and development industry and to academic researchers. In contrast to our following cases studies, EA efforts with NTDs have sought to improve the distribution or allocation of existing resources rather than to lead innovation in science or policymaking. Mobilizing a distinctive definition of neglect premised upon the epistemic superiority of their ‘view from the outside’, EAs also enact a broader measurement ethos aimed at strategic prioritization and cost-efficiency within philanthropy. Assuming the diminishing utility of both financial and knowledge investment, they identify underfunded, high-mortality diseases as a high-value area for philanthropic support to targeted populations (MacAskill, 2015; Todd, 2013). At the same time, they often question the soundness of their own metrics. Measuring neglect and effectiveness involves multiple re-categorizations, shedding light on the messy, contested character of philanthropic value creation.

## Case-study 2: Cultured meat and the valorization of uncertain futures

Another key area of EA intervention is ‘effective animal advocacy’ (Broad, 2018). Several EA organizations advocate ‘cultured meat’ as a way to minimize animal suffering. Cultured meat refers to the growing of meat in a bioreactor directly from animal cells through biomedical tissue-engineering techniques applied to food (Abrell, 2023; Post, 2012), a pursuit involving several university researchers and around 100 related start-ups since the early 2000s. Non-profit organizations and grant-giving bodies also advocate cultured meat, most notably New Harvest and the Good Food Institute, both based in the United States. This led to the production in 2013 of the world’s first laboratory-grown burger, as well as small-scale demonstration products such as sausages, meatballs, chicken nuggets and small steaks (O’Riordan *et al*., 2016, Stephens & Ruivenkamp, 2016). Cultured meat products were first commercialized in limited quantities in late 2020 in Singapore and have been available in the United States since 2023. While challenges remain to successfully commercialize them at volume – including making them cheap and sufficiently stabilized and standardized that they can be consistently produced at scale – the cultured meat project has been sustained by a set of promissory narratives about the moral cost of conventional meat production and animal farming, the value of a Silicon Valley-inspired ‘disruptive’ business model, and the power of technology to realize moral progress (Chiles, 2013; Jönsson, 2016; Mouat & Prince, 2018; Sexton *et al*., 2019; Stephens *et al*., 2019). Supporters have argued that cultured meat will cause less environmental damage than conventional meat, improve food safety by avoiding health risks related to animal-borne disease and antibiotic use, and allow the production of meat without killing animals. Consumed in vast quantities, it will cause a food revolution without the need to change the moral views of individuals. Consumers, the dominant framing of publics in this discourse, will spontaneously turn to cultured meat so long as it is as cheap, accessible and tasty as conventional products.

For our interviewees, the role of EA in redrawing the boundaries of cultured meat as both technoscientific project and community is clear: EAs shape debates about, funnel additional support for, and directly contribute to producing relevant expertise. Animal Charity Evaluators, an organization originally called ‘Effective Animal Activism’, was founded in 2012 by Eitan Fischer, then a philosophy graduate. Hosted initially by the Centre for Effective Altruism, it became an independent charity in 2013. Defining its mission as ‘to find and promote the most effective ways to help animals’ (Animal Charity Evaluators, 2024), it offers donation advice through online charity reviews, provides advocacy advice, and evaluates charity: Animal Charity Evaluators awarded New Harvest ‘Standout Charity’ status in 2015, and the Good Food Institute ‘Top Charity’ status in 2016 (Animal Charity Evaluators, 2017). Fischer, who went on to work for a plant-based and cultured meat company before co-founding his own, is one of several start-up founders associated with EA. Other EAs look to join companies or third sector groups. One interviewee in 2019 told us that a leading company in the field, Upside Foods, ‘has a ton of people in EA on staff’. Another suggested that 20–40 per cent of the cultured meat community were EAs. EAs also produce randomised control trials of potential consumer attitudes on cultured meat, with the aim to improve public acceptance (Anderson & Bryant, 2018). As one interviewee claimed, ‘a lot of Effective Altruists fund the NGOs – New Harvest, GFI – so they must make a huge impact on the community’. By investing attention, time and money in cultured meat, EAs assert its moral value, provide resources for the technology, and contribute to establishing its status as a credible business opportunity and technological project.

In comparison to the case of NTDs, assessing the future efficiency of cultured meat requires accommodating high technological and economic risk. EAs, while asserting the need for data-driven analysis in identifying causes to support, also insist that cultured meat is an early-stage technoscientific project with limited material accomplishments. Both Animal Charity Evaluators and EA group Sentience Politics emphasize the risky nature of investment in cultured meat and the partial ignorance resulting from the absence of robust empirical data on how it will be produced, what it will cost, or its potential environmental impact. In their report on New Harvest, Animal Charity Evaluators (2017) stated: ‘we think that developing cultured animal alternatives is a potentially high-impact way to influence the food system. It seems plausible that demand for conventional meat will decrease once cultured meat is put on the market’. At the same time, they noted: ‘we believe that our back-of-the-envelope calculation of their cost-effectiveness is too speculative’ (Animal Charity Evaluators, 2017). When responding to a Good Food Institute projection that making cultured meat competitive could take about a decade, they claimed: ‘we suspect that a decade might be somewhat optimistic, and our current estimate is that 10–70 years might be more realistic’. Similarly, Sentience Politics (2016) stated that ‘[a]ssessing the resource efficiency of industrial processes that don’t yet exist involves making many informed assumptions, many of which will later turn out incorrect’ before concluding that ‘cultured meat is expected to be significantly more resource-efficient than animal agriculture, especially when predictions of future meat consumption are taken into account’ (p. 3). Ultimately, both claims of partial ignorance and the possibility of accelerating returns justify risky investments in cultured meat.

EA’s philanthropic metric work is instrumental in demonstrating the efficiency of cultured meat as a means to reduce global suffering. For instance, comparing the need for, and effectiveness of interventions aimed at reducing both human and non-human suffering, Sentience Politics defended cultured meat as part of its aim to expand humanity’s moral consideration to all sentient beings beyond humans (Sentience Politics, 2016). Defining cultured meat as a neglected pathway to addressing animal suffering, one report reads: ‘cultured meat research has received very little attention so far, making it relatively easy to conduct basic research that may later prove catalytic to further development’ and ‘[t]his, in combination with its potentially extraordinary return on animal and human welfare in the long term, convinces us that accelerating cultured meat is a worthwhile investment at this time’ (Sentience Politics, 2016, p. 7). Animal Charity Evaluators (2018) considered that ‘the availability of cost-competitive cultured animal products has the potential to cause a massive decrease in the demand for non-cultured animal products’, insisting that estimates of future cultured meat availability ‘meaningfully affect the prioritization of animal advocacy interventions’. All the while acknowledging the speculative nature of the technology, EAs do not question the legitimacy of standard risk assessment tools or the heuristic value of the ITN criteria. Investment in cultured meat is also underwritten by judgements about what the good society should look like and who is best placed to bring it into being. It encodes a non-anthropocentric order in which beings are granted moral status based on their capacity to experience pain rather than the biological species to which they belong (see e.g. Singer, 2011). From this perspective, interventions aimed at animal and at human welfare are commensurable and can be assessed and ranked on one common scale.

EA has received ambivalent responses from engineers and entrepreneurs involved in cultured meat. One scientist explained: ‘I’m very uncomfortable with the amount of dogmatic thinking in EA … there’s a lack of humility … but I think that EA has brought in a lot of dollars, which has been good’. A start-up founder also raised concerns that ‘the way [EAs] do moral calculus is not just immoral and shady, but also uneducated and not well thought out’. Questioning the credentials and expertise of EAs, he noted: ‘this ends up with [EAs] who are like, ‘I’m going to form a company’. And it’s like, ‘what are your qualifications?’ … they have functioned in an environment which encourages participation, but it doesn’t require content’. Another interviewee, explaining how they lost confidence in EA practice, made a similar critique: ‘the problem with effective altruism in general is people sitting in a room and deciding unilaterally what’s the best use of funding money, when they’re not experts in the space … It’s extremely frustrating because I often see these individuals who … love thinking they know a whole lot about things that they’re really not that informed on, and making some little, tiny equation that supposedly justifies how good their spending is’. These critics see EAs as ‘dogmatic’, ‘uneducated’, and ‘not experts’: influential but self-selected figures, whose lack of technical training can lead to poor investment decisions and potentially threaten the credibility of the cultured meat project.

The cultured meat case shows how EAs assess the future profitability of cultured meat through its moral value, derived from its presumed efficiency to reduce animal suffering. Underlying EA metric work in this case is a commitment to the idea of commensurability between human and non-human suffering. EAs also mobilize technoscientific promises that are common in venture capital valorization, and they fund startups to advance research and development on cultured meat products. As with the NTDs case, entrepreneurs and scientists involved in cultured meat development contest EA interventions for their lack of relevant expertise. EAs themselves, meanwhile, produce ‘back-of-the-envelope calculations’ of the future moral and financial benefits that cultured meat could generate, seeking to establish at once EA expertise and cultured meat as valuable philanthropic opportunity.

## Case-study 3: AI safety and the securing of long-term value

AI safety, the aim to mitigate potential risks from advanced AI, is another key EA cause. A matter of ‘extremely high importance, high neglectedness and reasonable tractability’ according to EA organizations GiveWell and Open Philanthropy (Karnofsky, 2016b), AI safety involves theoretical mathematical models of hypothetical AI general or superhuman capabilities (Legg, 2008), machine learning techniques to ‘align’ the behaviour of AI systems with users’ preferences (Amodei *et al*., 2016), and global policy agendas for governing ‘frontier AI’ (UK Department for Science, Innovation and Technology, 2023). Prominent AI scientists such as Stuart Russell, a computer science professor at the University of California, Berkeley, have described it as the inclusion of basic engineering considerations to their discipline (Russell, 2019). More controversial claims have pointed to the ‘existential risks’ to humanity’s survival over the long-term that future AI could pose (Altman *et al*., 2024; Bostrom, 2014; Hawking *et al*., 2014). AI safety research has received substantial funding and public support in recent years, including through Elon Musk donating $10 million to fund ‘long-term research’ on AI risks (Russell *et al*., 2015), technology companies DeepMind and OpenAI pledging to ensure that ‘artificial general intelligence (AGI) … benefits ‘all of humanity’ (OpenAI, 2018), and the UK government launching in 2023 the Frontier AI Taskforce, a ‘start-up inside government’ to build ‘state capacity’ in AI safety (Frontier AI Taskforce, 2023).

In contrast with NTDs and cultured meat, the main challenge of AI safety, as defined by EA organizations, has been to produce relevant academic expertise (Todd, 2015). The ambition to develop academic subjects relevant to AI safety is evident in Open Philanthropy’s grant-making strategy. According to its website, between 2016 and 2023, the organization directed more than $270 million to AI research with a ‘safety’ focus, including $30 million to the then non-profit OpenAI, $211 million to the Center for Human-Compatible AI (CHAI) at UC Berkeley, and $14 million to the Machine Intelligence Research Institute (MIRI), a transhumanist^2^ organization launched in 2000 to prepare for the advent of a technological singularity (Yudkowsky, 2001; see Taillandier, 2021b). Interviewees generally insisted that AI safety was a contested research area. A graduate student in computer science described it as a topic that one would choose for moral, rather than scientific reasons: ‘for a while I didn’t agree with [EA] arguments about AI safety … because they seemed to conflict with what I knew about AI and machine learning’. As he explained, ‘what convinced me to work on AI safety was thinking more about population ethics’, which led him to reassess the moral value of future people’s welfare and reconsider his own career strategy on this basis. A computer science professor similarly described in moral terms his decision to shy away from a ‘fringe’ research topic: ‘maybe if I were a better person, I would be spending more time researching AI safety … [but] if I did, I would stop getting funding, and I wouldn’t be able to support that many students’. For those settling on AI safety as priority cause, the expected moral value of AI safety outweighed its material and symbolic costs.

EAs typically assess the importance and legitimacy of AI safety as a philanthropic cause and research through invoking their intuition about its high expected moral value. In 2011, Holden Karnofsky (2015), GiveWell co-founder and CEO of the Open Philanthropy Project, explained why he would not recommend offering a grant to support MIRI even though he agreed ‘with many of [its] most controversial views’ – notably ‘that [unfriendly AI] is an existential risk and that [friendly AI], if it could be created before [unfriendly AI], would be astronomically beneficial’ (Karnofsky, 2015). As in the NTD case, Karnofsky (2015) based his decision on an ‘outsider’ judgment about the institute’s capacity to contribute to the field: ‘my intuitions don’t matter much because I know so little about artificial intelligence research and other relevant issues; but when I look at the actions of the people who seem to me that they ought to know better … few explicitly endorse your mission and capability to follow through on it … [M]y view of your organization hinges heavily on the question of whether you have people who have rare insights, capabilities, and general rationality, and if in fact you have those things, you ought to be able to translate them into impressive achievements/affiliations’. In 2015, Open Philanthropy revised its decision about both MIRI and AI safety.

Open Philanthropy’s requalification of AI safety as priority area was the result of a ‘shallow investigation’ of AI risks consisting of informal discussions with researchers and investors who had publicly raised the alarm about global catastrophic risks (Open Philanthropy, 2015). Karnofsky explained how an Open Letter by the Future of Life Institute and the publication of Nick Bostrom’s *Superintelligence* (2014) had led him to the ‘new interpretation’ that ‘*there simply is no mainstream academic or other field (as of today) that can be considered to be “the locus of relevant expertise” regarding potential risks from advanced AI* … There is no one who I think clearly qualifies as an expert on such matters, and no one I’m aware of who has clearly put more thought into the relevant issues than Nick Bostrom or relevant staff at MIRI’ (Karnofsky, 2016c, p. 5, emphasis in original). Having previously ‘misjudged’ the importance of AI risks, he hoped that ‘next time a cause this potentially promising emerges’, he would ‘recognize it faster’ (Karnofsky, 2016c, p. 6). Others within the organization noted that neither expert surveys nor quantitative extrapolations from current progress constituted reliable sources to anticipate future breakthroughs in AI (Muehlhauser, 2015b). As in the cultured meat case, EAs primarily drew on entrepreneurial intuition to assess the urgency and importance of AI safety for a ‘hits-based’ approach to philanthropy (Karnofsky, 2016a).

AI safety remains largely debated within EA. Some EA philosophers advocated prioritizing AI safety on the basis of longtermism, the idea that ‘the long-term future of humanity matters more than anything else’ (Wiblin, 2017). Longtermism, they argued, required adjusting EA metrics, rather than departing from evidence-based philanthropy or a more general commitment to ‘the scientific method’ (MacAskill, 2019, p. 15; MacAskill, 2022; Ord, 2020).^3^ Longtermism was also, as William MacAskill (2019) insisted, ‘compatible with any empirical view about the best way of improving the long-run future’. Others, for instance Singer (2015), expressed some reservation on EA prioritizing existential risk mitigation over the fight against poverty (pp. 165–174). For our interviewees, whether AI safety should be prioritized also revealed tensions between ‘the elite of the movement [Ord, MacAskill, Karnofsky]’ and activists. A researcher at Open Philanthropy noted: ‘there is a little bit of a disconnect with the leadership of EA, having shifted in that direction and being more interested in [existential] risks … I think there is some tension around that, and maybe that feels out of touch with the rest of the EA community’. One interviewee, then a fellow of the Oxford-based Future of Humanity Institute led by transhumanist philosopher Nick Bostrom until 2024, explained how longtermism had played out in his own view: ‘[EA] was initially very much like, global health is this area where we’ve got … roughly crisp measures of what we can work on in terms of costs to save a life, so we can rank everything, we can have these top options like malaria or deworming’. Incorporating ‘astronomical’ (Bostrom, 2003) quantities of future welfare into EA calculations, as suggested by a longtermist perspective, ‘[weighed] against the malaria and deworming agenda' by making NTD interventions appear as lower impact strategies. At the same time, he acknowledged the speculative nature of the argument, hoping that ‘it’s not the fad of a weird longtermist cult, and we are just following the evidence wherever it leads us’. As in our previous cases, EAs acknowledge the epistemic difficulty of producing evidence.

This latter case further illustrates how EA’s metric work translates moral valuation of future welfare into funding decisions. EA interventions in AI safety support research in computer science, forecasting or ethics, both at leading academic institutions such as Berkeley and Oxford and at transhumanist organizations selected for their long-time involvement in the study of existential risk. As in the case of cultured meat, EAs typically acknowledge the speculative character of long-term predictions and the limits of existing expertise, including their own. Evident in longtermist calls to prioritize increasing the number and welfare of future people in philanthropic decisions, the main justification for funding AI safety is its potential moral ‘high reward’ rather than its strong ‘tractability’. The ambition to reshape the long-term future, as a researcher at the Oxford Future of Humanity Institute put it, also makes EA ‘the continuation of transhumanism in a much wider sense’. In line with computer scientists stating that social responsibility should be part of the very definition of AI, EA organizations seek to lead innovation in AI research and regulation, as well as to reshape the field as a whole.

## Discussion: Philanthropic metric work as technoscientific worldmaking

One of the most prominent forms of philanthrocapitalism today, EA philanthropy promises to improve the world by optimizing individual donations, career strategies, venture capital investment and research grant allocation. EA translates philosophical principles – for instance, the notion that the wellbeing of each person should be given equal consideration regardless of space, time or species – into a variety of ranking and ordering tools. EA’s philanthropic metric work involves a range of quantitative methods – such as RCTs, cost–benefit analysis, technology forecasting, market predictions and the application of what EAs called the ITN framework – and qualitative ones – such as interviews with AI experts and historical comparison to assess ‘highly uncertain’ but ‘plausible’ future technological risks (Muehlhauser, 2015b; Open Philanthropy, 2015). Metric work stabilizes definitions of well-being and need: in the fight against extreme poverty, EA interventions typically prioritize access to deworming treatments over public health infrastructure development. It also establishes commensurability across sites of intervention, allowing actions aimed at human and animal welfare to be ranked on the same scale – a process that Marion Fourcade (2016) has termed ‘ordinalization’. Finally, EA metrics reduce the uncertainty associated with contested actions, for instance when calculations about the extremely large potential value of AI safety for future generations overweigh the difficulty of assessing the long-term tractability of both AI risks and mitigation strategies. Acknowledging epistemic limitations and ignorance – both theirs and those of traditional experts – EAs produce evidence of expected value not in spite of moral and economic uncertainty, but on the basis of it. EA philanthropy, in this sense, illustrates the well-studied role of risk mitigation strategies and promissory narratives in technoscientific innovation (Beckert & Bronk, 2018; Borup *et al*., 2006). Through producing criteria and evidence of importance, neglect and effectiveness, and mitigating the uncertainty of risky investments, metric work constructs the world that EAs seek to change, as well as the means to do so.

EA philanthropy entails a familiar ‘economics of techno-scientific promises’ (Joly, 2010), whereby the moral imperative to improve human and non-human welfare justifies innovation. But where expectations of moral progress and profitability are often found to take part in the construction of technological inevitability, our cases show that EA metric work often fails to materialize objectivity and indisputability or to stabilize the horizon of a linear future. EAs hold different views on whose wellbeing matters the most and on the most appropriate ways to improve present and future global welfare. These contrasting views are especially evident in debates around cause prioritization, with some defending the ‘safe bet’ of supporting NTDs and others advocating AI safety as a ‘risk worth taking’ (Karnofsky, 2016a). This diversity of views, far from unacknowledged, is a core tenet of EA’s rhetoric and practice – as reflected in Open Philanthropy’s commitment to ‘worldview diversification’ (Karnofsky, 2016d). Changes in EA priorities, rather than a straightforward shift away from the ‘low-hanging fruit’ of anti-malaria bednets to the ‘high-risk, high-reward’ projects of cultured meat and AI safety or a logical consequence of its philosophical commitments – for instance the broadening of moral concerns from humans regardless of their location in space, to non-human animals, and finally to future people, human or non-human^4^ – is best understood as the result of struggles between conflicting moral and economic expectations.

Our cases also draw a more nuanced picture of philanthrocapitalism to the one that critics have commonly portrayed. EA, while contributing to what McGoey and Thiel (2018) have called the ‘sanctification of the super-rich’, also effectively turns average-income donors into philanthropic experts in their own right. As the cases of cultured meat and AI safety make clear, EA draws upon and enacts entrepreneurial imaginaries that endow investors with special foresight and intuition – reiterating the mainstream liberal assumption that market actors can foresee the changing parameters of value creation and lead progress through risk-taking (Boenig-Liptsin & Hurlbut, 2016). More generally, EA metrics, based on apparently rigid and decontextualized criteria of importance, tractability and neglectedness, in fact largely accommodate the subjective preferences, intuitions and value judgements of ‘self-taught’ individuals. In part through a critique of traditional knowledge authorities and through performing ‘strategic ignorance’ (McGoey, 2012b), EAs assert their authority to steer innovation and moral progress: self-appointed experts, they insist, are more likely to make morally valuable choices than academics. Further elements of EA practice – such as its grassroots component, self-description as a social movement, and rhetoric of (partial) privilege acknowledgment – reflect its ambition to democratize philanthropy and achieve social change through investments and donations unimpeded by traditional political oversight or corporate governance. In line with ‘alternative philanthropy’ (Silver, 2007) and ‘philanthrolocalism’ (Beer, 2015), EAs reclaim the right for every potential donor in the two per cent global top-earners to define and address world problems in their own terms.

More than an instrument or a tool for enforcing a unified, consistent view of the good, technoscientific production is an integral part of EA’s attempt to make philanthropic expertise accessible to every donor. EAs challenge the monopoly of traditional experts and professional communities over the definition of what constitutes optimal investment and qualified philanthropic knowledge, either through tailoring existing objectifying tools to their needs, or through developing their own expertise. In the NTD case, they identify global health problems and priorities in their own way, contesting the categories in use in global development, public health and biology research. In cultured meat and AI safety, by contrast, they seek more direct involvement in the making of technoscience, either through founding cultured meat startups or through seeking to make a ‘foundational contribution’ to AI and machine learning (Whittlestone, 2015). The continuing relevance of traditional knowledge hierarchies, credentials and ambitions not only constraints EA claims to expertise, but it also puts crucial limits to its stated ambition to radically transform and democratise philanthropy through the aggregation of individual moral preferences and decisions. Ultimately, EA’s imaginary of social change through the rule of experts challenges the vanguard, entrepreneurial model of philanthropy led by visionary and benevolent technoscientists.

## Conclusion

Philanthropy performs a complex ideological function. It materializes the moral superiority of economic elites, hides the role of donations in tax avoidance, increases global inequalities and reinforces the view that capitalism can benefit the greater number. Endorsed by prominent technology billionaires and philosophers in recent years, EA has become increasingly visible. While most scholarship discusses its utilitarian commitments or reiterates long-standing critiques of philanthropy more generally, our paper brings emphasis to the concrete practices through which EAs translate moral claims into action. Through comparing three case-studies – NTDs, cultured meat and AI safety – we fleshed out some of the ambiguities, contestations, identity-work and localized interactions that shape EA practice. By studying EA in the making, we provide empirical support to the claim that it contributes to fostering existing configurations of social and market power as privileged ways to social change.

We showed that philanthrocapitalism is a complicated ideological project, one that entails egalitarian aspirations and decentralized knowledge practices alongside justifications of the socioeconomic *status quo*. A heterogeneous set of practices, EA’s philanthropic metric work entails measuring how a singular donation increases the welfare of a particular category of benefactors (the global poor, non-humans, or future people), recasting what counts as neglected in global health policy, or incorporating entrepreneurial intuition into risk-benefit analysis. It is also an operation in sociotechnical worldmaking, whereby the tools to assess social change are themselves a particular modality of philanthropy. By developing its own risk assessments, categorizations and rankings, EA *de facto* contests dominant forms of philanthropic and academic expertise. Empowered with rationalization techniques such as the ITN framework, each donor can decide not only whether and where to give, but also which areas of philanthropic intervention are legitimate, and which are not. Well, aligned with other forms of philanthrocapitalism, EA enacts dominant entrepreneurial imaginaries, whereby gifted individuals are seen as best positioned to lead social change, knowledge production and technological innovation. But EA also furthers a more traditional view of philanthropy as production of expertise. Ultimately seeking to reshape technoscientific domains by remaking academic fields from within, as in the case of AI safety and computer science, EA philanthropy entails both challenging traditional philanthropic expertise and reiterating its high-modernist ambition to change the world from above.

By shifting the focus from metrics to metric work, we showed that philanthropic instruments express conflicting moral views. Metrics, in our cases, act not only as neutralizing tools that enable philanthropic action under uncertainty but also as highly politicized knowledge interventions based on different understandings on what constitutes the world’s most pressing problems and the most efficient ways to address them. Seeking to maximize the value of philanthropic actions through supporting, reshaping, or fostering technoscientific projects with high expected market and moral value, EAs remake promissory narratives and channel resources towards controversial projects – be they upscaling bednet distribution as a way to address NTDs, or embracing the financial promises of the ‘AI fetish’ (Kampmann, 2024). Effectively blurring the boundary between philanthropic and technoscientific worldmaking, the study of EA sheds light on the changing repertoires of philanthropic value creation, and the ambitions of the technology elite to remake the world in their own image.

## Notes

1 Our argument is compatible with the view that EA philanthropy is otherwise aligned with dominant imaginaries of race and gender, among others. Our use of gendered pronouns makes visible the unmistakable masculine character of the EA movement.

2 Transhumanism aims to fundamentally change the human condition through technoscience. On the relationship between EA, existential risk and transhumanism, see Davidson (2022), Srinivasan (2015) and Torres (2023).

3 MacAskill (2022) draws in part on Nick Bostrom’s claim that ‘existential risks’ – unlikely risks to humanity’s survival or potential for future flourishing, such as extreme technological risks related to AI – should be granted specific epistemic status and prioritized in global policy (Bostrom, 2002, 2013, 2014) as well as on long-standing ethical discussions of future moral obligations, most notably associated with the work of moral philosopher Derek Parfit. See also Beckstead (2013).

4 This idea is captured by the notion of expanding ‘moral circle’ mentioned by our interviewees and developed by moral philosopher and EA advocate Peter Singer (2011).
